# Enhancement of Temperature Fluorescence Brightness of Zn@Si Core-Shell Quantum Dots Produced via a Unified Strategy

**DOI:** 10.3390/nano11113158

**Published:** 2021-11-22

**Authors:** Mohammad S. Almomani, Naser M. Ahmed, Marzaini Rashid, M. K. M. Ali, H. Akhdar, O. Aldaghri, K. H. Ibnaouf

**Affiliations:** 1 School of Physics, Universiti Sains Malaysia, Minden 11800, Penang, Malaysia; Mohammadmomani36@yahoo.com (M.S.A.); marzaini@usm.my (M.R.); 2 Department of Physics, College of Science, Imam Mohammad Ibn Saud Islamic University (IMSIU), Riyadh 11623, Saudi Arabia; hamofarog@yahoo.com (M.K.M.A.); odaghri@gmail.com (O.A.); kheo90@gmail.com (K.H.I.)

**Keywords:** ZnSiQDs, electrochemical etching, photoluminescence, quantum confinement, core–shell

## Abstract

Despite many dedicated efforts, the fabrication of high-quality ZnO-incorporated Zinc@Silicon (Zn@Si) core–shell quantum dots (ZnSiQDs) with customized properties remains challenging. In this study, we report a new record for the brightness enhancement of ZnSiQDs prepared via a unified top-down and bottom-up strategy. The top-down approach was used to produce ZnSiQDs with uniform sizes and shapes, followed by the bottom-up method for their re-growth. The influence of various NH_4_OH contents (15 to 25 µL) on the morphology and optical characteristics of ZnSiQDs was investigated. The ZnSiQDs were obtained from the electrochemically etched porous Si (PSi) with Zn inclusion (ZnPSi), followed by the electropolishing and sonication in acetone. EFTEM micrographs of the samples prepared without and with NH_4_OH revealed the existence of spherical ZnSiQDs with a mean diameter of 1.22 to 7.4 nm, respectively. The emission spectra of the ZnSiQDs (excited by 365 nm) exhibited bright blue, green, orange-yellow, and red luminescence, indicating the uniform morphology related to the strong quantum confinement ZnSiQDs. In addition, the absorption and emission of the ZnSiQDs prepared with NH_4_OH were enhanced by 198.8% and 132.6%, respectively. The bandgap of the ZnSiQDs conditioned without and with NH_4_OH was approximately 3.6 and 2.3 eV, respectively.

## 1. Introduction

Inorganic semiconducting colloidal nanoparticles with uniform morphology and narrow size distribution have been widely investigated owing to their fundamental scientific importance and diverse practical applications [1]. Amongst all the recently studied semiconductor nanostructures, the colloidal Si quantum dots (SiQDs) became increasingly attractive due to their non-toxicity, abundance, and tunable bandgap energies [2]. On top of that, SiQDs can be used for a wide range of applications, such as optoelectronics, solar cells, biomedical devices, and light-emitting diodes (LEDs) [2]. Generally, both chemical and physical methods are used to synthesize SiQDs. Physical methods (bottom-up and top-down approaches) include (1) plasma synthesis: the diameter of these particles is between 2 and 8 nm, and the emission covered all visible spectrum, but this method needs low pressure or high temperature, thereby increasing the cost of equipment and fabrications; (2) laser ablation: the advantage of this method is the clean technique, and the average particle size is between 20 and 500 nm; to prepare a small size (2–3 nm) the process must be carried under low pressure, hence increasing the economic burden. The top-down chemical approach includes (3) electrochemical etching. It is the most famous method to synthesize SiQDs due to the user-friendly equipment used and facile preparation, but the size and shape of SiQDs cannot be controlled. The bottom-up approach involves (4) the reduction of the Si halides and Zintl phase oxidation. The size of SiQDs prepared by this method is between 5 nm and 3 μm and requires high temperature and low pressure. One of the significant benefits of porous silicon (PSi) is its ease of production. PSi is primarily synthesized by electrochemical dissolution of Si pieces in a hydrofluoric acid solution. PSi can be created by electrochemical etching or anodization in a hydrofluoric solution. In terms of equipment and chemicals utilized, the electrochemical-etching process is a straightforward and cost-effective experimental setup [3,4]. The electrochemical-etching process is preferred to produce Si nanoparticles. In this procedure, the Si wafer (acts as a cathode) is immersed in an electrolyte made of an HF–ethanol solution, wherein the platinum (Pt) or graphite rod acts as an anode [5]. In the past, the electrochemical-etching technique has widely been used to produce colloidal SiQDs [6]. The room-temperature visible light emission from nanoscale PSi was first demonstrated by Canham [6], which was attributed to the effect of quantum confinement. It has been reported that electrochemically etched PSi obtained from p- or n-type Si wafers in various solvents (such as methanol, water, ethanol, acetone, and toluene) upon ultrasonication can form abeyance of SiQDs. The abeyance constitutes a broad size distribution in the range of micrometers to nanometers. In addition, it is possible to achieve tiny SiQDs in a size range of 2–11 nm by controlling the sieve (20 nm) together with prolonged ultrasonication of PSi [7]. It is established that the indirect bandgap of bulk Si is transformed into a direct or quasi-direct one when the size of the sample becomes comparable to or below the bulk exciton Bohr radius (~5 nm), wherein the quantum yield (QY) can reach up to 90% due to the quantum size effect [7]. The optical and electrical attributes of SiQDs can be improved by modifying the surface morphology (sizes and shapes of particles), chemical bonding, and via doping with the transition metals [8]. Additionally, the incorporation of ZnO into the PSi was shown to significantly enhance the room-temperature visible photoluminescence (PL) emission intensity as much as seven-fold [9]. The bandgap energy displays an inverse correlation with the size of the SiQDs, and the emission wavelength reveals a blue shift as the size of the SiQDs reduces, which can be ascribed to the effect of strong quantum confinement [9]. It is worth noting that the previous report on the electrochemically etched synthesis of SiQDs revealed the difficulty of attaining uniform sizes [10]. To surmount this limitation, ammonium hydroxide (NH_4_OH) was used for preparing spherical nanoparticles such as Si [11], titanium dioxide [12], silver [13], magnetite [14] and ZnO by the assembly of tiny nanoparticles [13].

In addition, strong bases, including KOH and NaOH, plus weak bases, such as NH_4_OH, were used to grow tiny core–shell nanoparticles [15]. Despite many dedicated efforts, the production of high-quality ZnO incorporated core–shell SiQDs (ZnSiQDs) with customized properties remains challenging. This motivates us, in this work, to prepare ZnSiQDs in acetone using electrochemical etching of a Si wafer following the protocols referred to in [16]. The influence of different amounts of NH_4_OH addition (15 to 25 µL) on the morphology and optical (room temperature absorbance and PL emission) characteristics of the as-prepared ZnSiQD suspension were evaluated. The sizes and optical properties of these QDs were shown to significantly depend on the NH_4_OH contents, indicating the feasibility of tailoring the room temperature PL properties by adjusting the amount of NH_4_OH [17].

Previously, various metal elements (including Cr^+3^ and V_2_O_5_) were incorporated into the PSi film by immersing them in a solvent to improve the PL of the PSi. Furthermore, the PSi surface was coated with various metals of varying thicknesses to alter the overall properties. It was also assumed that by doping SiQDs with various transition metals, one could alter their microstructures and morphologies, thereby improving their optical proper [18,19,20]. It was proposed that the incorporated transition metals act as a host substratum in the PSi, improving the visible PL emission traits [20]. It is renowned that each of these metallic elements possessing 18 core electrons and 6 valence electrons in the 3p shell shows several characteristics when doped; the number of electrons differs to the degree that they can turn into the active outermost 3d electrons, hence participating in the optical transitions with relaxed selection rules [21]. In an attempt to enhance optical characteristics, high-quality PSi integrated with Zn powder was synthesized in a single step. The transition metal Zn was chosen because it belongs to the transition metals that served as the host substrate [20], it dissolves directly in HF, and it combines with O to form ZnO. The optical properties of colloidal SiQDs derived from PSi are primarily due to the effects of quantum confinement, ligands, and various surface chemistries, as well as surface defects, which produce new energy levels in the bandgap region, facilitating radiative recombination of (e^−^-h^+^) pairs [22,23].

**Figure 1:**
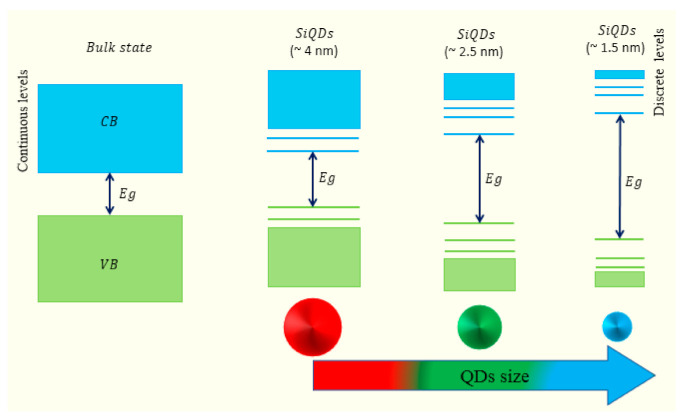
Schematic depiction of how the change from the bulk to the QDs impact the CB, VB levels, and Eg in QDs.

The intrinsic states may be reduced as the size of the quantum dots (QDs) and the number of atoms in the dots increase. When the relative position of these intrinsic states in the band diagram becomes lower than the edge state, there is a loss of expected emission. The offset between energy levels (surface states and band position) that determine optical properties is one of the important parameters. The fluorescence effect differs between small and large QDs. It can be seen that the offset between surface states and LUMO is greater for small QDs than for large QDs. In this case, the emission wavelength is determined by the excitation wavelength. Moreover, as the size of the dots increases, such a reliance degrades, and bandgap-mediated transitions start increasing. Excitation-independent emission behavior is caused by the obstructing of surface state-based transitions and the appearance of some functional groups. It has been shown that QDs such as carbon dots (CDs) with amino-rich surface groups exhibit less dependence on excitation wavelength due to surface state passivation [23] Bands are established in standard semiconductors such as Si by the partnership of neighboring energy levels of a very large number of atoms and molecules. Nevertheless, as the particle size approaches the nano-size and the amount of atoms and molecules drops rapidly, the number of intersecting energy levels reduces, allowing the band to widen, and the energy levels become discrete and quantized. Because QDs are so small, they have a larger energy gap between the valence and conduction bands than bulk states. The quantum confinement and discrete energy levels effect are two main features for QDs. As a result, the characteristics of quantum dots vary with their size, and their excitations are confined in all three-dimensional space. The major feature of a quantum dot that describes the relationship between QD size and the wavelength of light they generate is confinement energy [24]. Figure 1 illustrates the effect of size-changing from bulk to quantum size on the electronic level and the bandgap value. The special characteristics of QDs, which are caused by their unusually high surface-to-volume ratios, clarify why these nanocrystals can produce different colors based on the size of particles. As the particle size decreases, the energy levels in the conduction band (CB) and the valance band (VB) become discrete (quantized), as exemplified in Figure 1. Thereby, so much energy is required to excite the particle, and more energy is dissipated when the quantum dot returns to its state of relaxation. If the size of quantum dots is changed, they will produce any color of light within the same material [25].

## 2. Materials and Methods

### 2.1. Materials

High-purity (99.99%) chemical reagents of hydrogen fluoride (HF), ethanol (C_2_H_5_OH), zinc (Zn) powder, acetone (C_3_H_6_O), NH_4_OH (28.0 to 30.0%) were purchased from Sigma-Aldrich Chemical Co. (St. Louis, MI, USA). n-type Si (100) (thickness and resistivity in the range of 355–405 µm, and 0.002–0.005 Ω.cm, respectively) were used to synthesize the PSi incorporated with Zn (ZnPSi) and colloidal Si quantum dots (SiQDs) combined with zinc (ZnSiQDs following the method prescribed in [9,16].

### 2.2. Preparation of ZnPSi and Suspension ZnSiQDs in the Presence/Absence of NH_4_OH

N-type (100) Si wafer was cut via a diamond cutter in rectangles of dimensions 1.5 cm × 2.5 cm^2^. The Radio Corporation of America (RCA) process was used to clean and remove the native oxide from the Si chips [16]. In the ring-etching cell (made of Teflon), the Zn powder of mass of 0.17 g was added in a mixture of HF and ethanol at a volume ratio of 1:1. To form the ZnPSi film, n-type Si (100) acts as an anode, while platinum (Pt) wire acts as a cathode and is immersed in the HF/ethanol solution. The etching cell was illuminated via a tungsten lamp through the etching method conducted at various current densities (5 to 45 mA/cm^2^ with the step of 5 mA/cm^2^); the optimum properties of ZnPSi were achieved when the etching current density was 5 mA/cm^2^. After electrochemical etching was completed, a circular disk of brown color was produced on the Si substrate. In addition, an orange-red light was emitted from the brown disk under excitation wavelength (UV light). The electropolishing was utilized to separate the ZnPSi from the n-Si substrate. A high current density value was applied; brown pieces of ZnPSi film were collected by centrifugation for the HF/ethanol (at 1000 rpm to 5 min). The resultant brown pieces were ultrasonicated in acetone for 60 min to generate a grey solution via filtration and centrifugation (at 1500 rpm for 30 min); colloidal ZnSiQDs in acetone solution was obtained. The previous steps formed the top-down method of production of the ZnSiQDs. The following steps include the bottom-up approach to the re-growth of the ZnSiQDs. First, different amounts of NH_4_OH (15, 20, and 25 µL) were added to the colloidal ZnSiQDs suspended in acetone and incubated in a dark place for 72 h. Then, under the ultra-violet (UV) light influence, various colors were emitted from suspension ZnSiQDs with NH4OH. Finally, to fix the size of colloidal ZnSiQDs with NH_4_OH, 1 µL of polyvinylpyrrolidone (PVP) was added to 40 µL of DI water and mixed with 20 mL colloidal ZnSiQDs with NH_4_OH in acetone; the mixture was stirred for 30 min. As-prepared samples were characterized at room temperature.

### 2.3. Characterization of ZnPSi and ZnSiQDs Colloids

X-ray diffractometer (Bruker D8 Advance, AXS GmbH, Karlsruhe, Germany) was utilized to study the crystallinity of the ZnPSi films, with the Cu K1 line of wavelength 1.54 Å being used. The morphology of the colloidal ZnSiQD suspension was analyzed using an energy-filtered transmission electron microscope (EFTEM, Libra 120, Zeiss GmbH, Oberkochen, Germany). UV–vis–NIR absorption spectroscopy (Cary 5000, Agilent, CA, USA) was used to record the absorption spectra of the colloidal ZnSiQD suspension. The photoluminescence spectrometer attached with HPC-2 collimated light source was used to measure the emission spectra of the samples (at the excitation wavelength, λ_exc_ = 325, 365, 390, and 425 nm). The field emission scanning electron microscope (FESEM, FEI Nova SEM 450, FEI Company, Hillsboro, OR, USA) was used to image the morphology of the samples. The trace elements in the ZnSiQD suspension were detected using the energy-dispersive X-ray (EDX) spectrometer. A Bruker optics FT-IR spectrometer model (Tensor 27, Bruker Optics Ltd., Coventry, UK) was utilized to capture Fourier transform infrared (FTIR) spectra. The survey spectrum of X-ray photoelectron spectroscopy (XPS) was measured using a monochromatic Al Ka supply prepared Kratos Axis Ultra DLD device (Kratos Analytical Ltd., Manchester, UK).

## 3. Results and Discussion

### 3.1. Morphology and Structure of ZnPSi

**Figure 2:**
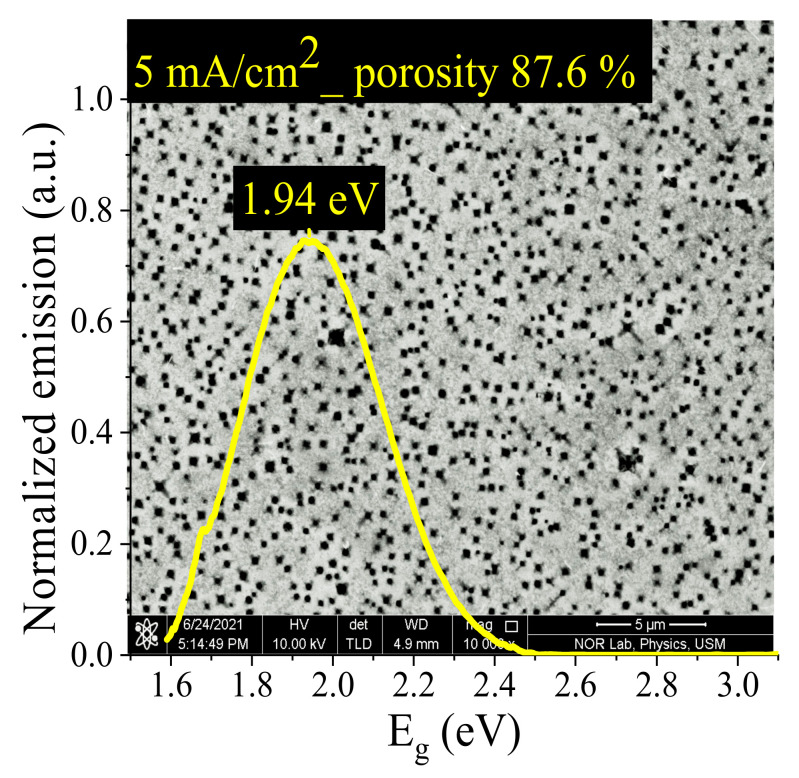
FESEM morphology combined with PL of the PSi etched at 5 mA/cm^2^.

Figure 2 illustrates the FESEM image together with their PL of ZnPSi etched at the current density of 5 mA/cm^2^; a high number of pores were achieved at 5 mA/cm^2^. Weight measurements can easily estimate porosity, defined as the fraction of vacancy within the PSi layer. The porosity of the ZnPSi was determined using the gravimetric relation [26]:

(1) $$Porosity\;\left(\%\right)=\frac{m1-m2}{m1-m3}\times 100\%$$

where *m*_1_ is the mass of the samples before etching, *m*_2_ is the mass of the samples after etching, and *m*_3_ is the mass of the samples after removing the PS layer with KOH solution. The PSi thickness can be calculated by Equation [26]:

(2) $$Thickness=\frac{m1-m3}{\rho S}$$

where the *ρ* and *S* are the Si density and ZnPSi area, respectively. The area of ZnPSi was 3.14 cm^2^ at a diameter of 2 cm, and the Si density was 2.33 g/cm^3^. The PL is proportional to the porosity; when the porosity increased, the PL shifted to short-wavelength due to a decrease in the bandgap, which was a result of the reduced crystallite size [27,28].

**Figure 3:**
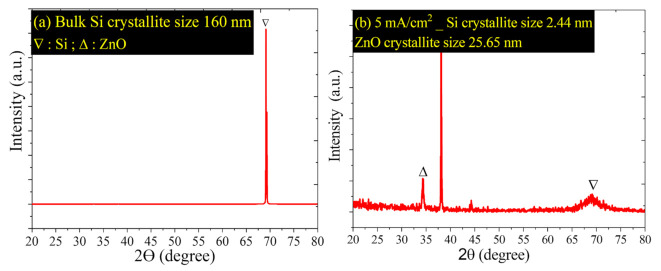
XRD analysis for (**a**) bulk Si; (**b**) ZnPSi at etching current density of 5 mA/cm^2^.

Figure 3 shows the XRD analysis for ZnPSi etched at 5 mA/cm^2^ at diffraction angles (2θ) of 20–80°. The crystallite size was calculated by using the Scherrer equation [28]:

(3) $$D=\frac{K\lambda }{\beta *\cos\left(\theta \right)}$$

*D*: grain size, *K*: constant (0.9), λ: XRD wavelength, *θ*: Bragg angle, and *β*: full width, half maximum (FWHM) for XRD peaks. Figure 3a shows the crystallite size Si before and after etching of 160 nm and 2.44 nm, respectively, due to the change in the morphology of Si after etching. Figure 3b reveals the generation of ZnO and Si nanostructure; the crystallite size of ZnO is 25.65 nm. The sharp crystallite size was reduced because of the generation of pore layers with high porosity and small wall thicknesses. Referring to Figure 1 and the result in Figure 2, the PL of ZnPSi is related to the quantum confinement effect because the Si crystallite size is less than 10 nm [29,30].

**Figure 4:**
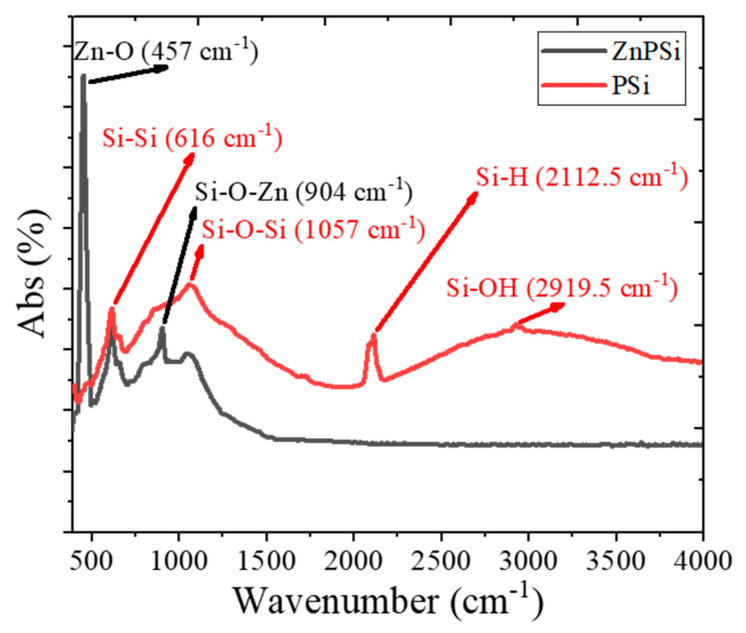
FTIR spectra of Psi and ZnPSi.

Figure 4 illustrates the FTIR spectrum of native (control) PSi and PSi incorporating Zn (ZnPSi). The control PSi has peaked at 616 cm^−1^, 1083 cm^−1^, 2113.5 cm^−1^, and (3000–4000 cm^−1^), which refer to bonds of Si-Si, Si-O-Si, Si-H, and Si-OH, respectively, while ZnPSi has peaked at 457 cm^−1^, 615 cm^−1^, and 903 cm^−1^, which refer to bonds of Zn-O, Si-Si, and Si-O-Zn, respectively [31,32,33]. The peaks centred at 1057, 2112.5, and 2921 cm^−1^ of ZnPSi vanished due to the generated ZnO shell, which prevents oxidation of the SiQDs; also, the new sharp peaks at 457 cm^−1^ and 904 cm^−1^ refer to the stretching band of ZnO because of the ZnO shell produced around SiQDs. It was reported that the addition of Zn powder to the electrolyte during the electrochemical etching could produce ZnO on the surface of SiQDs. The structure of the compound semiconductor is core and shell; the wider bandgap semiconductor (shell) acts as a potential barrier for the narrower bandgap (core) [34,35]. However, the production of Zn-O and Si-O-Zn can demonstrate that Zn^+2^ ions were successfully doped into the inner SiQDs layer and that the SiQDs layer was fully coated [35].

**Figure 5:**
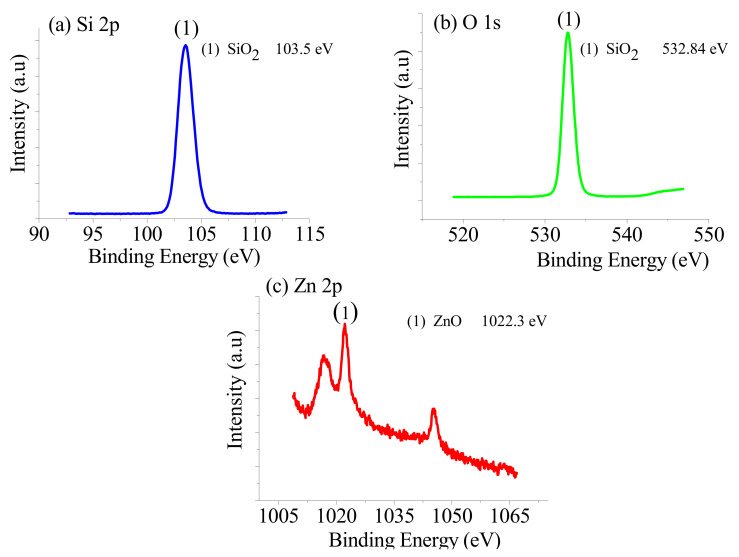
The XPS profile of the ZnPSi for (**a**) Si2p (103.5 eV), (**b**) O1s (532.84 eV), and (**c**) Zn2p (1022.3 eV).

**Figure 6:**
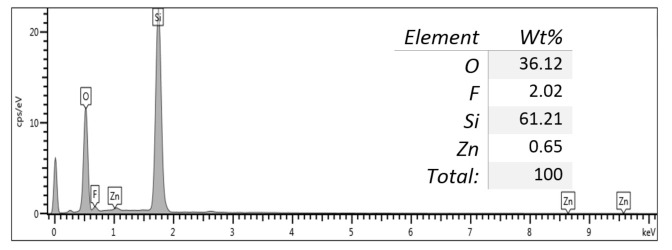
EDX images of the prepared ZnPSi with 0.17 g of included Zn.

Figure 5 shows the XPS profiles of the ZnPSi, which verified the presence of elements such as Zn, F, Si, and O. The existence of Zn and O on the surface and in the channels of the porous layer led to the formation of Zn-O and Si-O linkages. Thus, the O1s peak can be fitted with two components, including Zn-O and Si-O linkages [36,37]. Figure 6 depicts the EDX spectral analysis of the prepared ZnPSi with 0.17 g of Zn. The elemental traces (weight %) in the ZnPSi are shown in the included table attached to the image.

### 3.2. Morphology and Structure of ZnSiQDs

**Figure 7:**
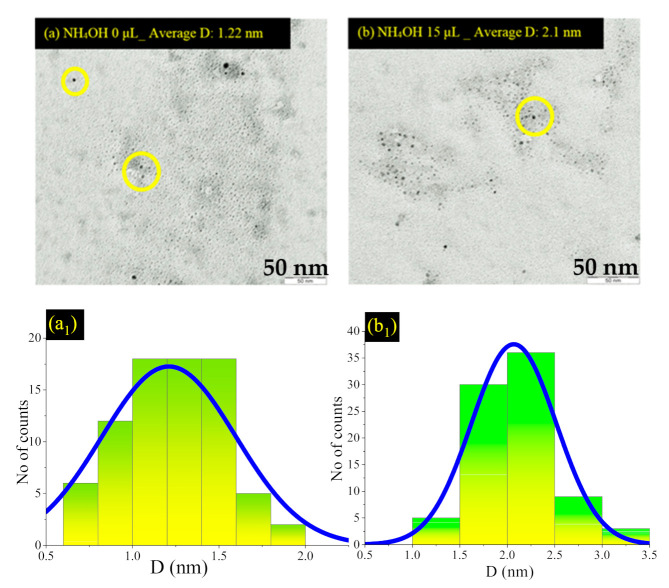

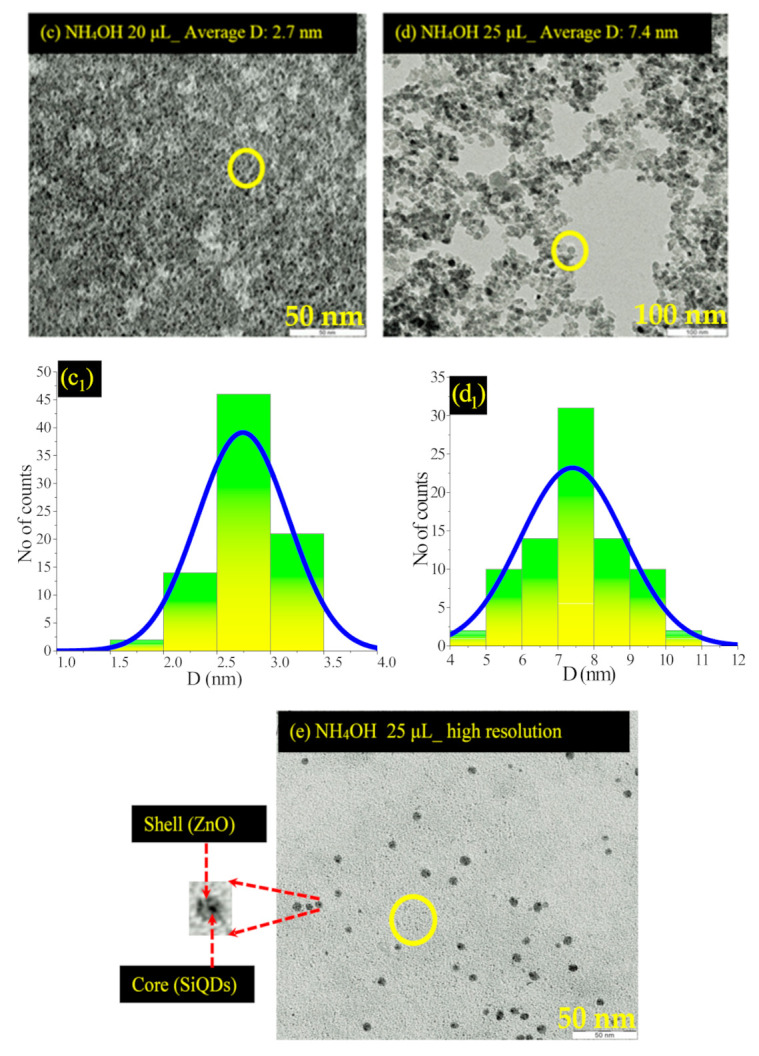
(**a**–**d**) EFTEM images of the colloidal ZnSiQD suspension in acetone prepared with NH_4_OH of 0, 15, 20, and 25 µL, respectively. (**a_1_**–**d_1_**) particle size distributions of the colloidal ZnSiQDs (20 mL) suspended in acetone with NH_4_OH of 0, 15, 20, and 25 µL, respectively. (**e**) NH_4_OH: 25 μL high resolution.

Figure 7a–d show the EFTEM micrographs, and Figure 7(a_1_–d_1_) show particle size distributions of the colloidal ZnSiQDs (20 mL) suspended in acetone without (a) and with NH_4_OH (b,c, and d) of 15 µL, 20 µL, and 25 µL, respectively. Samples prepared without and with NH_4_OH showed the nucleation of spherically shaped ZnSiQDs (yellow circle) with the corresponding mean size of 1.22 nm and 2.1, 2.77, and 7.4 nm. The dimensions of QDs enlarged with the addition of NH_4_OH, indicating the strong influence of NH_4_OH on the aggregation, nucleation, and growth of the tiny ZnSiQDs. In short, the sizes and shapes of the ZnSiQDs were significantly affected by the NH_4_OH, wherein the QDs size distribution became more uniform, and the inter-particle separation was reduced. The inclusion of NH_4_OH in the colloidal ZnSiQD suspension enabled the re-growth of the smaller particles to form the chain by producing a core centre [38]. In this study, the size of ZnSiQDs was minuscule, between 1.22 and 7.4nm, so to determine the major shape of these particles, an EFTEM image was completed again for the largest particles with low concentrations to obtain an image with high resolution; the particles have an approximately spherical shape, as shown in Figure 7e. The tiny particles (a yellow circle of Figure 7e) unioned to generate the large particle, as shown in the image on the left of Figure 7e. That particle has two regions; the first part is the core (blackish point), the second part is the shell around the core. Thus, this image supports the hypothesis of the generated core/shell between SiQDs and ZnO.

### 3.3. Optical Characteristics of ZnSiQDs

**Figure 8:**
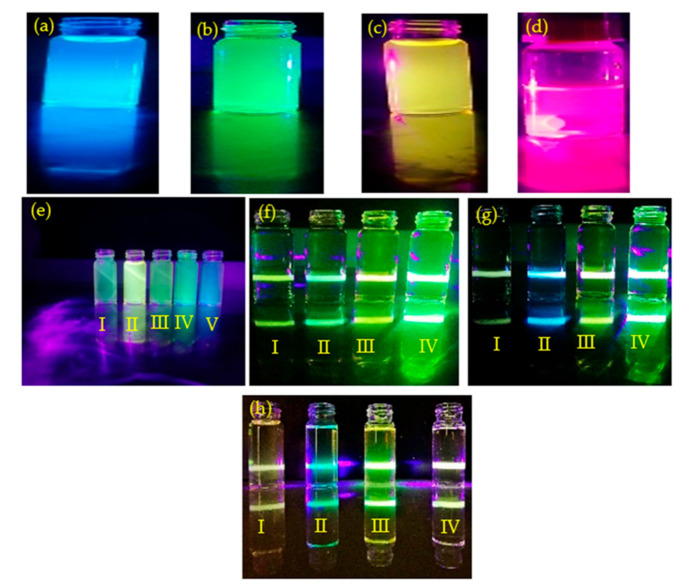
Luminescence from colloidal ZnSiQDs containing various amounts of NH4OH under different excitation (**a–d**) added with 15 µL, 20 µL, 25 µL, and 30 µL of NH_4_OH excited at 365 nm, respectively; (**e**) bottles I, II, III, IV, and V with 17 µL, 25 µL, 19 µL, 20 µL, and 15 µL of NH_4_OH excited at 365 nm added, respectively; (**f**) bottles I, II, III, and IV with 17 µL, 19 µL, 22 µL, and 20 µL of NH_4_OH excited at 410 nm added, respectively; (**g**) bottles I, II, III, and IV with 17 µL, 15 µL, 22 µL, and 20 µL of NH_4_OH excited at 410 nm added, respectively; (**h**) bottles I, II, III, and IV with 16 µL, 15.5 µL, 21.5 µL, and 14 µL of NH_4_OH excited at 410 nm added, respectively.

Figure 8a–f illustrate fluorescence from the ZnSiQDs prepared with different amounts of NH_4_OH under the excitation wavelength of 365 nm and 425 nm, respectively. The obtained colloidal ZnSiQDs showed strong green, blue, and yellow-orange emissions. Furthermore, the luminescence brightness of these ZnSiQDs was enhanced with the increase in NH_4_OH contents. This indicated the significant role of NH_4_OH that controlled the QDs size by producing the core center to re-grow these QDs from tiny sizes, thereby achieving the uniform-sized QDs with narrow size distributions. Moreover, the excitation wavelengths’ independence of the luminescence brightness verified the uniform size distribution of the majority of the suspended QDs. For example, the sample (20 mL) containing colloidal ZnSiQDs with 20 µL of NH_4_OH added exhibited the same green emission brightness under the excitation of 365 nm and 425 nm, respectively (Figure 8b, and f(IV) or g(IV)). The sample (20 mL) containing colloidal ZnSiQDs with 15 µL of NH_4_OH added displayed a similar blue emission brightness when excited with 365 nm and 425 nm, respectively (Figure 8a,g(II)).

**Figure 9:**
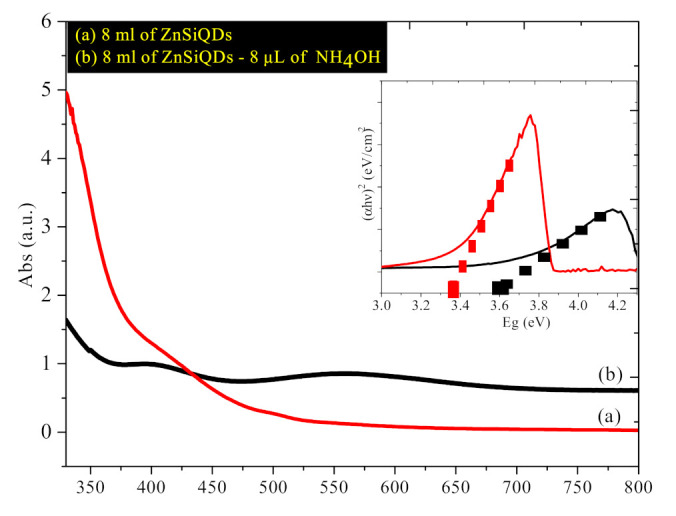
ZnSiQD suspension in acetone prepared (**a**) without and (**b**) with NH4OH (Inset: Tauc plot for the bandgap estimation.

Figure 9a,b show the UV–Vis–NIR absorption spectra of the colloidal ZnSiQD suspension (20 mL) in acetone without and with NH_4_OH (20 µL) addition. The absorption intensity was improved by 2.5 times, and the peak was shifted to a longer wavelength (from 300 nm to around 330 nm), indicating the formation of larger particles or an increase in the density of the particles [39,40]. The inclusion of NH_4_OH led to the rise in the growth of the nanoparticles, wherein the ZnSiQDs prepared with NH_4_OH showed higher absorbance than the one prepared without NH_4_OH. The inset displays the Tauc plot used to evaluate the optical bandgap energy (E_g_) of the ZnSiQDs. For both samples, the absorbance was dropped as the wavelength was increased. The higher absorption intensity for the ZnSiQDs containing NH_4_OH can be attributed to the higher transition probability because of the higher electronic density of states in the QDs. The value of E_g_ for the ZnSiQDs without and with NH_4_OH inclusion was approximately 3.6 eV and 3.35 eV, respectively. The observed reduction in the value of E_g_ for the ZnSiQDs containing NH_4_OH can be ascribed to the QDs’ size enlargement.

**Figure 10:**
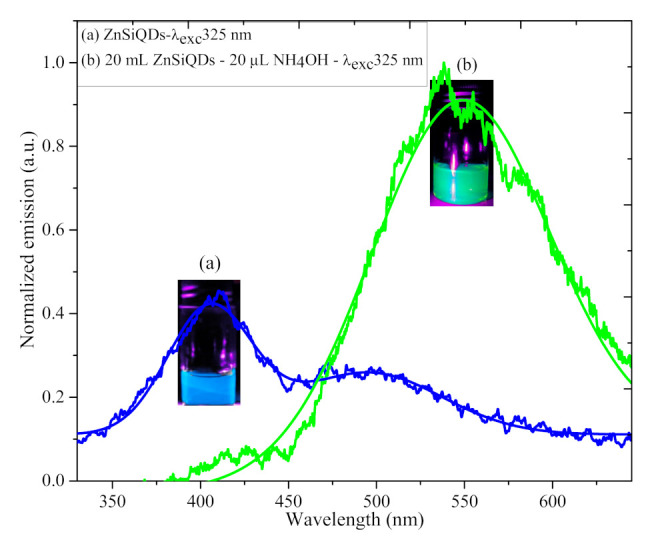
PL spectra (excited at 325 nm) of the colloidal ZnSiQD suspension in acetone synthesized (**a**) without and (**b**) with NH_4_OH (20 µL). The bottles in the inset show the corresponding blue and green visible fluorescence observed by the naked eyes.

Figure 10 shows the PL spectra (excited at 325 nm) of the colloidal ZnSiQD suspension (20 mL) in acetone without and with NH_4_OH (20 µL) inclusion, indicating the visible emission in the blue and green region. The PL spectra of the ZnSiQDs without and with NH_4_OH exhibited a prominent fluorescence emission peak at 411 nm (blue) and 539 nm (green), respectively. Referring to the ZnSiQDs without NH_4_OH, the observed intense emission peaks of the ZnSiQDs at 411 that originated from the core consisted of pure SiQDs [41], while the peak at 539 nm was related to the defects in the ZnO shell [42,43]. The ZnSiQDs containing NH_4_OH showed a weak peak at 411 nm and a strong peak at 539 nm. The difference in peak intensity after the addition of NH_4_OH was due to the formation of a ZnO shell around SiQDs core. Due to the SiQDs surface passivation by ZnO, the emission from the core was damped or hindered. In addition, the excess amount of NH_4_OH could also affect the fluorescence emission from ZnSiQDs, leading to a red-shift in the peak emission wavelength from blue (411 nm) to green (539 nm). The growth of NH_4_OH-activated ZnSiQDs led to an increase in the particle size, narrowing the bandgap accompanied by a red shift in the emission peak.

**Figure 11:**
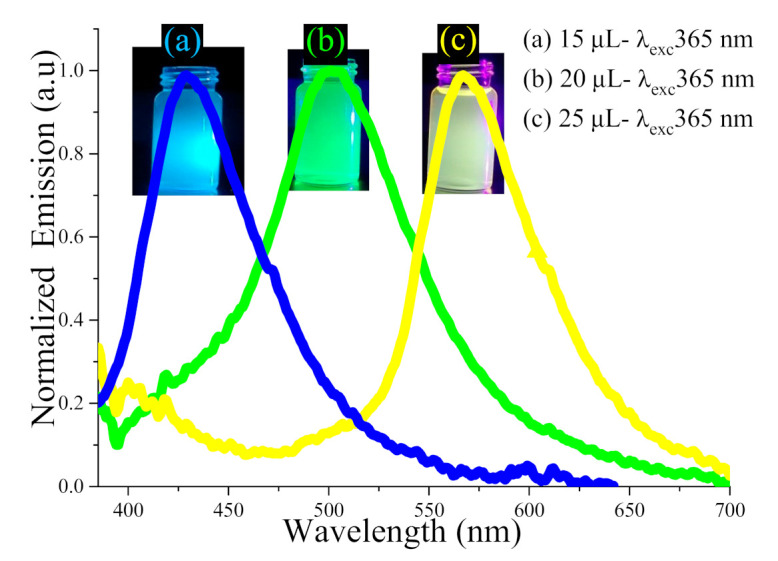
PL spectra (excited at 365 nm) of the colloidal ZnSiQDs synthesized with NH_4_OH of (**a**) 15 µL, (**b**) 20 µL, and (**c**) 25 µL.

**Figure 12:**
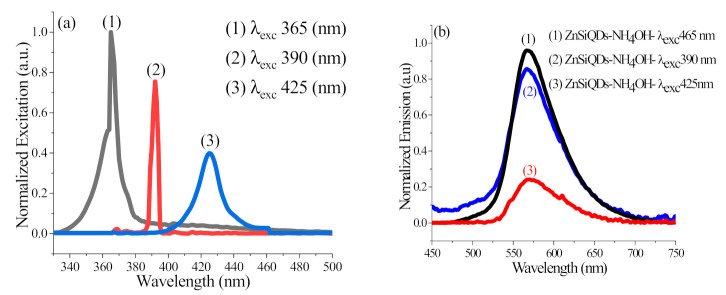
(**a**) Excitation spectra of source at excitation peak of (1) 365 nm, (2) 390 nm, and (3) 425 nm. (**b**) PL spectra of the colloidal ZnSiQD suspension in acetone containing 25 µL of NH_4_OH excited at the wavelengths of (1) 365 nm, (2) 390 nm, and (3) 425 nm.

Figure 11 depicts the PL spectra of the colloidal ZnSiQD suspension in acetone (under 365 nm excitation) synthesized with different amounts of NH_4_OH (15 to 25 µL), wherein the bottles in the inset show the corresponding blue, green, and yellow visible fluorescence observed by the naked eyes. The color of colloidal ZnSiQD suspension became darker when the NH_4_OH content was increased from 15 µL to 20 µL to 25 µL, where each sample showed a sharp (intense) emission peak under 365 nm excitation. This indicated the vital role of NH_4_OH in altering the morphology of the ZnSiQDs. Table 1 shows the sensitiveness of the emission peak wavelength and the corresponding spectral full width at half the maximum of the ZnSiQDs on the variation of the NH_4_OH content. This observed difference in the emission spectral properties of the three samples was attributed to the schism of the particles in the suspension due to activation via NH_4_OH. With the increase in NH_4_OH contents, the particle size was increased, generating short bonds between the tiny ZnSiQDs and enhancing the nucleation and growth rates. The strong fluorescence emission from ZnSiQDs containing 15 µL of NH_4_OH originated from the pure SiQDs, where the amount of NH_4_OH was insufficient for the SiQDs surface passivation by the ZnO shell [41]. Conversely, for the ZnSiQDs containing 20 and 25 µL of NH_4_OH, the emission peaks were related to defects in the ZnO crystal (oxygen vacancy) that was generated as the shell structure on the SiQDs’ surface due to the abundance of NH_4_OH and passivation of the SiQDs’ core. Besides, the variation in the emission peak position of the ZnSiQDs with NH_4_OH contents can be attributed to an alteration in the optical bandgap energy because of the quantum-size effect. The narrow emission spectrum implied the same (uniform) QD size in all samples [44], wherein the presence of NH_4_OH enabled the re-growth of the ZnSiQDs to the same size. To confirm this claim, the colloidal ZnSiQD suspension was further tested at different excitation wavelengths (365, 390, and 425 nm) as shown in Figure 12b.

Figure 12a shows excitation spectra of the source at an excitation peak of (1) 365 nm, (2) 390 nm, and (3) 425 nm, while Figure 12b illustrates the emission spectra of the colloidal ZnSiQD suspension with 25 µL of NH_4_OH added and excitation at various wavelengths. Table 2 shows the sensitivity of the emission peak wavelength of the corresponding spectral full width at half-maximum on the excitation wavelength variation. The emission peak position is independent of the excitation wavelength changes, indicating the existence of uniform-sized QDs in the suspension [18] or potentially a surface-state-related emission rather than the emission from the ZnSiQDs’ core. The emission intensity of the ZnSiQDs excited at 365 nm and 390 nm was almost the same, indicating their similar bandgap energy. However, the emission intensity of the ZnSiQDs excited at 425 nm was reduced five times, implying that the bandgap energy of the QDs was greater than excitation energy [45]. Figure 12a shows that the lowest intensity of the excitation source was at a wavelength of 425 nm, which is less than 40% of the excitation wavelength at 365 nm; therefore, it contains a small number of photons compared to other excitation sources. Because of this, the emission density decreases by a massive amount because the excitation source includes a few photons.

**Figure 13:**
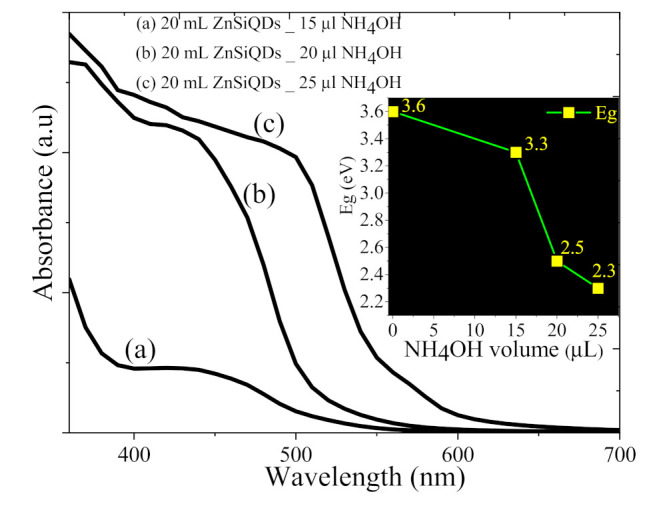
UV–vis absorbance of the colloidal ZnSiQD suspension in acetone synthesized with NH_4_OH of (**a**) 15 µL (**b**), 20 µL, and (**c**) 25 µL.

Figure 13 illustrates the UV–Vis absorbance of the colloidal ZnSiQD suspension in acetone synthesized with different amounts of NH_4_OH (15, 20, and 25 µL). The inset shows the NH_4_OH content-dependent variation in the optical bandgap energy of the ZnSiQDs. The value of bandgap energy was decreased from 3.6 to 2.2 eV with the increase in NH_4_OH contents from 15 to 25 µL, respectively. This drop in the bandgap energy value can be attributed to the generation of many OH^−^ and NH^4+^ from the higher volume of NH_4_OH, allowing for the growth of large ZnSiQDs [46].

### 3.4. Mechanism of ZnSiQDs Formation with NH_4_OH

**Figure 14:**
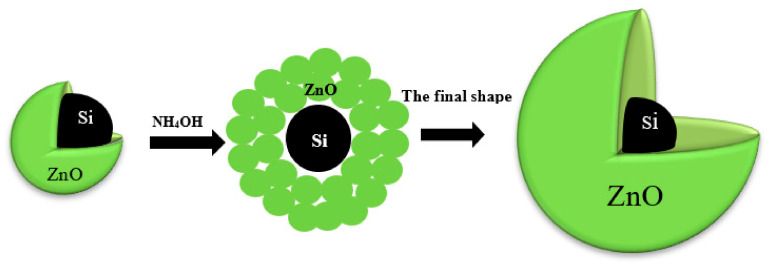
The schematic diagram for the mechanism of NH_4_OH influence on the ZnO shell.

Figure 14 presents the mechanism of NH_4_OH influence on the ZnO shell where the additive NH_4_OH is adsorbed into the ZnSiQDs. When NH_4_OH was added to the colloidal ZnSiQDs in acetone, it was dissociated into NH_4_^+^ and OH^−^. (Zn(NH_3_)_4_)^+2^ and Zn(OH)_2_ or (Zn(OH)_4_)^+2^) were produced due to the reaction of Zn^+2^ with NH_4_^+^ and OH^−^, respectively. The chemical reactions can be inferred through the following pathways [47]:

Path I: Zn^+2^ + 2OH^−^ → Zn(OH)_2_

Path II: Zn^+2^ + 4OH^−^ → Zn(OH)_4_^−2^

Path III: Zn^+2^ + 4NH_4_^+^ → Zn(NH_3_)_4_^+2^

The unstable nature of Zn(OH)_4_^−2^, Zn(OH)_2_, and Zn(NH_3_)_4_^+2^ enabled Zn(NH_3_)_4_^+2^ to react with OH^−^ via the chemical pathway [47]:

Path IV: Zn(NH_3_)_4_^+2^ + 2OH^−^ → ZnO + 4NH_3_ + H_2_O

The produced Zn(OH)_4_^−2^ congregates in the solvent, and ZnO served as the core of new aggregates while the surface generally contained Zn^+2^ and OH^−^. The size of the aggregates was increased due to the association of more Zn^+2^ and OH^−^ via the following. The chemical paths 5 and 6 summarize the last proposal [48]

Path V: Zn(OH)_4_^+2^ → ZnO + H_2_O + 2OH^−^

Path VI: Zn(OH)_2_ + 2OH^−^ → Zn(OH)_4_^+2^

With the increase in NH_4_OH contents, the number of NH_4_^+^ and OH^−^ was increased, thereby increasing the number of ion aggregates to produce the ZnO shell with Zn^+2^ and OH^−^ as the surface bonds. Consequently, the ZnO nanocrystalline shell grew along the *z*-axis due to its high-energy polar planar orientation, thereby producing nanorods [47]. This argument was supported by both EFTEM and FESEM images which showed spherical ZnSiQDs, indicating the growth of a ZnO nanocrystalline shell in different directions due to the presence of NH_4_OH as a complexing agent to shift ZnO preferential growth orientation.

## 4. Conclusions

A new record for the improvement of room-temperature brightness (blue, green, and orange-yellow) of colloidal ZnSiQD suspension in acetone is reported for the first time. Such colloidal ZnSiQDs were synthesized using a combination of top-down and bottom-up approaches. The synergy between these two methods enabled the production of these QDs with uniform sizes and shapes together with their re-growth. The inclusion of various amounts of NH_4_OH (15 to 25 µL) into the colloidal ZnSiQD suspension was shown to play a significant role, altering the overall morphology and optical properties of the ZnSiQDs. The formation of the ZnO shell around the SiQDs core via surface passivation due to the activation of NH_4_OH was responsible for improving the optical traits of the colloidal ZnSiQDs, especially the room-temperature visible luminescence. Using a mechanism with different chemical reaction pathways, it was argued that NH_4_OH served to grow the ZnSiQDs by an assembly of tiny particles to produce larger particles or re-grow the ZnO shell surrounding the SiQDs. The optical attributes of the ZnSiQDs were remarkably improved. The emission-peak wavelengths were independent of the excitation wavelengths and strongly dependent on the NH_4_OH contents, indicating the nucleation of QDs with a uniform size distribution. The colloidal ZnSiQDs exhibited a broad range of visible emissions in the blue, green, and orange-yellow region, indicating their effectiveness for the tandem solar cell and liquid laser applications. It is worth evaluating the effect of time on the growth process, which may elucidate more benefits of NH_4_OH-activated ZnSiQD development for functional applications. Future tasks will be focused on utilizing these QDs in rainbow solar cells.

## Figures and Tables

**Table 1:** Dependence of the emission peak wavelength (λpeak) and the corresponding spectral full width at half the maximum ZnSiQDs on the variation of the NH_4_OH content.

NH_4_OH (µL)	λ_peak_ (nm)	FWHM (nm)
15	428	68
20	501	87
25	567	80

**Table 2:** Dependence of the emission peak wavelength and the corresponding spectral full width at half-maximum ZnSiQDs on the excitation wavelength changes.

λ_exc_ (nm)	λ_peak_ (nm)	FWHM (nm)
425	567	70
390	567	57
365	567	62
